# A TiO_2_ nanorod and perylene diimide based inorganic/organic nanoheterostructure photoanode for photoelectrochemical urea oxidation†

**DOI:** 10.1039/d3na00294b

**Published:** 2023-11-09

**Authors:** Jasmine Bezboruah, Devendra Mayurdhwaj Sanke, Ajay Vinayakrao Munde, Palak Trilochand Bhattad, Himadri Shekhar Karmakar, Sanjio S. Zade

**Affiliations:** a Department of Chemical Sciences, Centre for Advanced Functional Materials, Indian Institute of Science Education and Research (IISER) Kolkata Mohanpur Nadia 741246 West Bengal India sanjiozade@iiserkol.ac.in

## Abstract

Visible light-driven photoelectrochemical (PEC) urea oxidation using inorganic/organic nano-heterostructure (NH) photoanodes is an attractive method for hydrogen (H_2_) production. In this article, inorganic/organic NHs (TiO_2_/PDIEH) consisting of a *N*,*N*-bis(2-ethylhexyl)perylene-3,4,9,10-tetracarboxylic diimide (PDIEH) thin layer over TiO_2_ nanorods (NRs) were fabricated for the PEC urea oxidation reaction (UOR). In these NHs, a PDIEH layer was anchored on TiO_2_ NR arrays using the spin-coating technique, which is beneficial for the uniform deposition of PDIEH on TiO_2_ NRs. Uniform deposition facilitated adequate interface contact between PDIEH and TiO_2_ NRs. TiO_2_/PDIEH NHs achieved a high current density of 1.1 mA cm^−2^ at 1.96 V_RHE_ compared to TiO_2_ NRs. TiO_2_/PDIEH offers long-term stability under light illumination with 90.21% faradaic efficiency. TiO_2_/PDIEH exhibits a solar-to-hydrogen efficiency of 0.52%. This outcome opens up new opportunities for inorganic/organic NHs for high-performance PEC urea oxidation.

## Abstract 2

Visible light-driven photoelectrochemical (PEC) urea oxidation using inorganic/organic nano-heterostructure (NH) photoanodes is an attractive method for hydrogen (H_2_) production.

## Graphical abstract

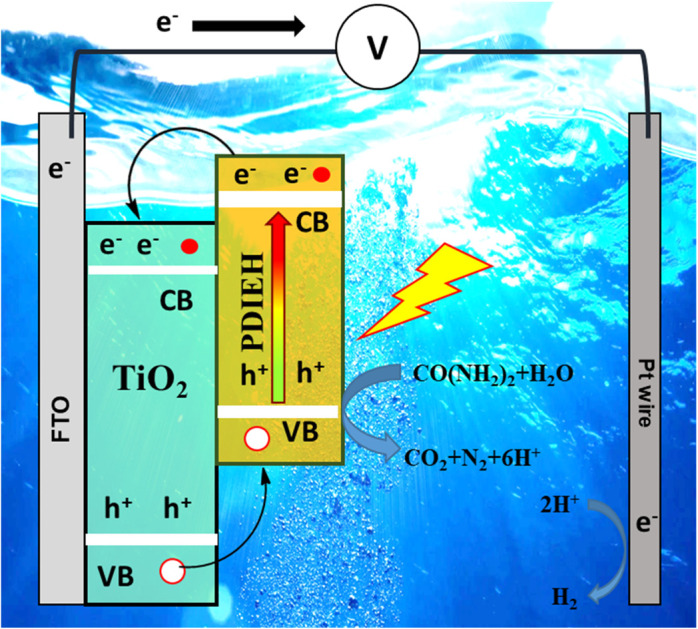

## 1. Introduction

Human society mainly uses exhaustible energy sources, like natural gas, natural oil, and coal.^1^ The combustion of these fuels produces environmental pollutants as a by-product, thus creating an environmental threat. Significant research efforts are being devoted to the development of clean and renewable energy sources as a replacement for traditional energy sources. In addition, urea is widely used as a significant nitrogen source in industry and agriculture.^2^ Urea is produced in large quantities from bio-waste and is widely used in the chemical industry.^3^ Urea from agriculture, industries, and domestic waste enters fresh water and acts as a pollutant. Treating urea in wastewater is essential due to the rising water pollution caused by urea discharge.^3,4^ Urea-containing wastewater can produce green hydrogen energy and reduces environmental pollution through the urea oxidation process. The process of producing hydrogen gas from urea-containing wastewater is promising from the standpoint of sustainable energy. This H_2_ energy is considered a potential fuel with high efficiency and no emission of harmful chemicals.^5,6^ The photoelectrochemical (PEC) urea oxidation reaction (UOR) has been proven to be an effective approach for H_2_ generation with low overpotential and energy input.^7,8^ Theoretically, the urea electrolysis voltage is only 0.37 V_RHE_.^9^ This makes PEC urea oxidation a vital method for H_2_ generation. UOR not only generates H_2_ but also reduces environmental pollution.

In 1972, the discovery of TiO_2_ electrodes as a photoelectrocatalyst by Fujishima and Honda opened the door for PEC studies.^10^ Later, extensive research on TiO_2_ in different forms, like TiO_2_ nanorods (NRs),^11^ nanotubes,^12^ and nanoparticles,^13,14^ as photocatalysts was carried out. Cho *et al.* reported branched TiO_2_ NRs for PEC H_2_ production.^15^ Niu *et al.* reported corrugated nanowire TiO_2_ as a versatile photoanode for PEC alcohol and water oxidation.^16^ Cho *et al.* reported codoping TiO_2_ nanowires with tungsten and carbon for enhanced PEC performance.^17^ Hwang *et al.* reported a TiO_2_/BiVO_4_/SnO_2_ triple-layer photoanode with enhanced PEC performance.^18^ Duan *et al.* reported a TiO_2_ nanowire/microflower photoanode modified with Au nanoparticles for efficient PEC water splitting.^19^ Park *et al.* reported photocatalytic UOR on TiO_2_ in water and urine.^20^ In addition to increasing the conductivity, TiO_2_ has been doped and blended with various metals^21–23^ and non-metals.^24–26^ To functionalize doped/undoped TiO_2_, various organic semiconductors have gradually emerged as beneficial candidates for designing TiO_2_ NH photoanodes.^27–29^

Perylene diimide (PDI) derivatives have received a lot of attention as organic semiconductors (OSCs)^30,31^ in the fields of fluorescent solar collectors,^32^ organic photovoltaics (OPVs),^33^ and organic field-effect transistors (OFETs).^34^ PDI derivatives possess excellent thermal and optical stability and good carrier transport properties.^35,36^ The relatively facile and reversible reduction process of PDI derivatives is important in many applications.^37^ The relatively simple and reversible reduction of PDIs is important in many of these applications. They are widely used in basic studies on photoinduced energy and electron-transfer processes because of their facile reductions, combined with their easily identifiable excited states, anions, and dianions *via* absorption spectra.^30,31^ Due to their fused-ring structures and strong intermolecular forces, they are poorly soluble in organic solvents. *N*-Alkyl substitution improves the solubility of PDIs in various organic solvents and enables the integration of PDI derivatives into NHs.^30^ To improve the photocatalytic activity of NHs, Zhang *et al.* reported NH composites of various PDI derivatives with TiO_2_.^38^ A TiO_2_ nanotube and a PDI were combined in an organic/inorganic heterojunction for PEC water splitting.^39^

**Scheme 1:**
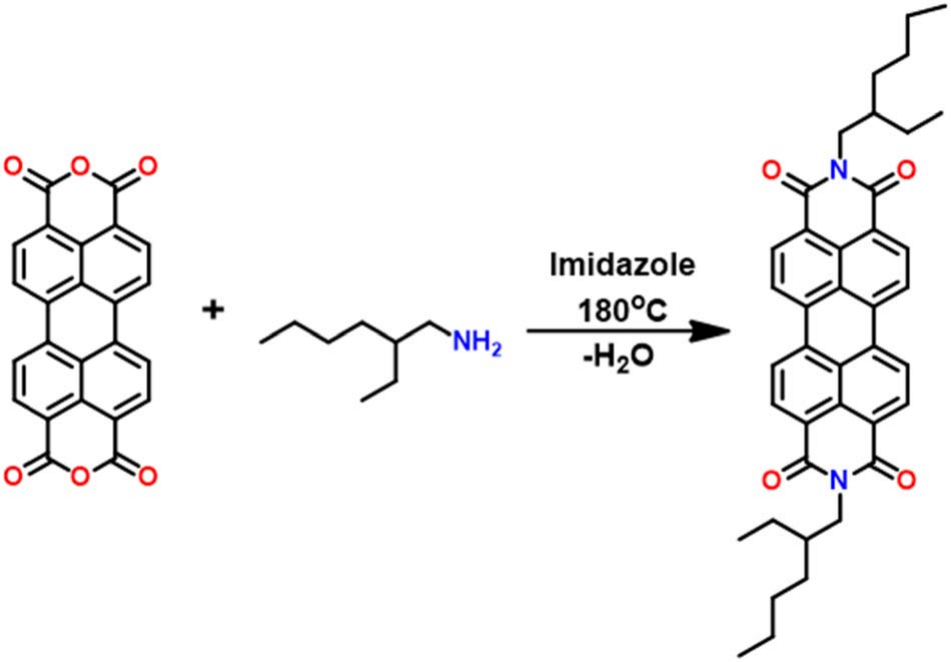
Synthesis of *N*,*N*-bis(2-ethylhexyl)perylene-3,4,9,10-tetracarboxylic diimide (PDIEH).

This paper focuses on the engineering of inorganic/organic NHs with *N*,*N*-bis(2-ethylhexyl)perylene-3,4,9,10-tetracarboxylic diimide (PDIEH) anchored on TiO_2_ NRs (represented as TiO_2_/PDIEH henceforth (Fig. S3†)). To synthesize PDIEH, commercially available PDA was used as the starting material and modified at an anhydride functional group with 2-ethylhexylamine (Scheme 1). The PEC properties of TiO_2_/PDIEH NHs towards PEC urea oxidation reaction (UOR) were investigated using various electrochemical measurements. A stability test was done by performing a chronoamperometry experiment. The morphological study was done by field emission scanning electron microscopy (FE-SEM).

## 2. Results and discussion

**Fig. 1:**
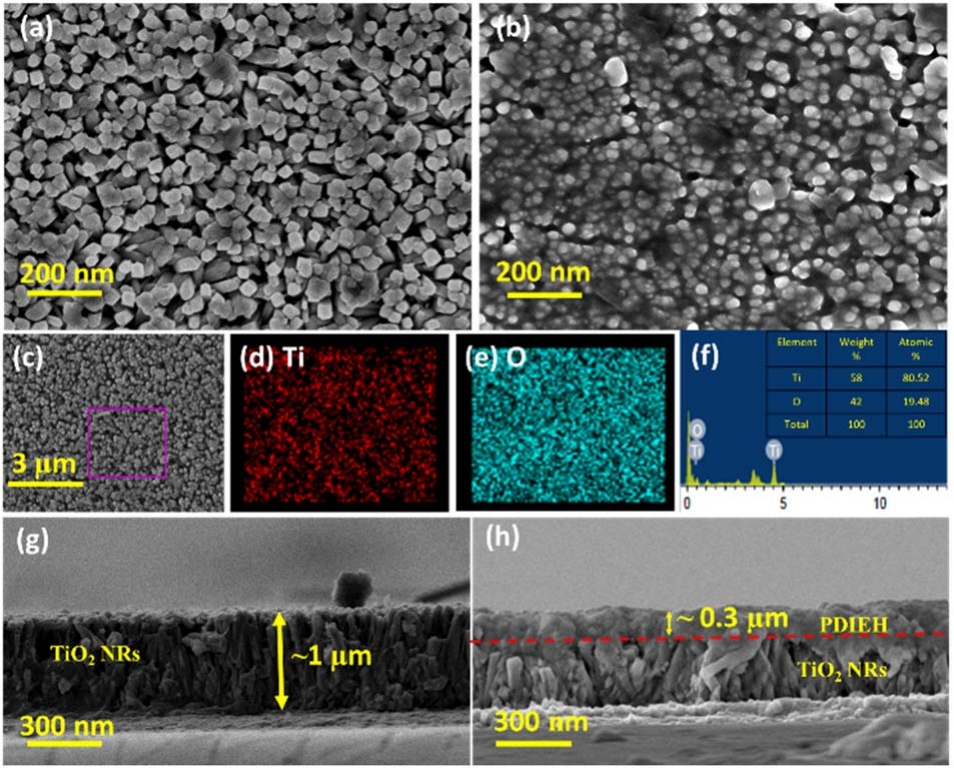
FESEM images of (a) TiO_2_ NRs and (b) TiO_2_/PDIEH NHs. (c) SEM image of TiO_2_ NRs for EDS measurement and corresponding element mapping of (d) Ti and (e) O. (f) EDX spectrum of TiO_2_ NRs. Cross-section FESEM images of (g) TiO_2_ NRs and (h) TiO_2_/PDIEH NHs.

The FESEM images of the TiO_2_ NRs reveal uniformly distributed TiO_2_ NRs grown vertically on the FTO substrate with a rectangular surface (Fig. 1a). The thin coating of PDIEH over TiO_2_ NRs can be seen in the FESEM images of the TiO_2_/PDIEH NHs (Fig. 1b). Element mapping showed the even distribution of Ti and O elements in TiO_2_ NRs (Fig. 1c–e). Furthermore, the energy-dispersive X-ray (EDX) spectrum of TiO_2_ NRs (Fig. 1f) showed Ti and O elements, confirming the successful formation of TiO_2_ NRs. Fig. 1g shows the cross-sectional FESEM image of the TiO_2_ NRs, which reveals an average size of ∼1 μm. The thick coating of PDIEH of ∼0.3 μm on TiO_2_ NRs is seen in the cross-sectional image of TiO_2_/PDIEH NHs with a strong interface between TiO_2_ NRs and PDIEH (Fig. 1h). Fig. S4a and c† show the top-view and cross-sectional FESEM images of TiO_2_/PDIEH NHs with the ∼0.6 μm thick PDIEH layer and Fig. S4b and d† show the top-view and cross-sectional FESEM images of TiO_2_/PDIEH NHs with the ∼0.8 μm thick PDIEH layer. Fig. S5a† shows the PXRD pattern of powder PDIEH, revealing its crystalline nature. A comparison of the X-ray diffraction (XRD) patterns for both photoanodes (TiO_2_ NRs and TiO_2_/PDIEH NHs) indicates the formation of NHs (Fig. S5b†). The XRD pattern of TiO_2_/PDIEH NHs shows additional peaks around 10° (enclosed in a red rectangle) corresponding to PDIEH. The Raman spectrum of PDIEH showed multiple peaks located at 1084 cm^−1^ (C–H bending vibrations), 1301 cm^−1^ (ring stretching), 1380 cm^−1^ (ring stretching), 1454 cm^−1^ (ring stretching), 1570 cm^−1^ (C=C stretching), 1587 cm^−1^ (C=C stretching), and 1612 cm^−1^ (C=C stretching), matching well with reported values (Fig. S4c†).^40,41^

**Fig. 2:**
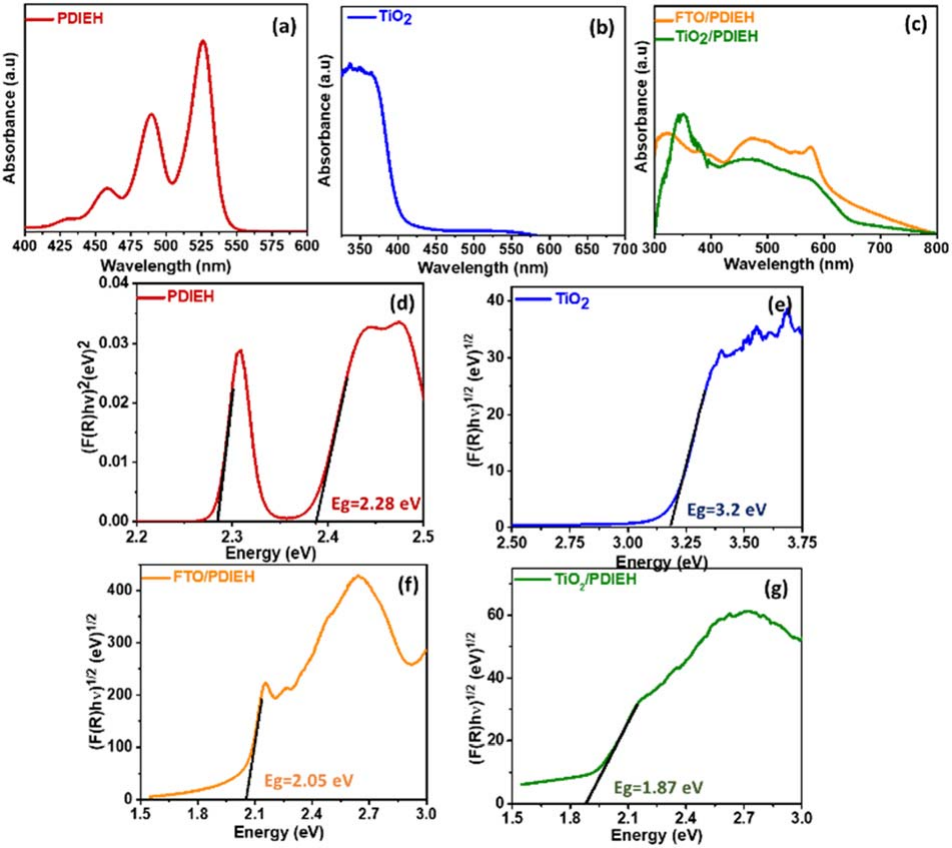
Absorbance spectra of (a) PDIEH in CHCl_3_, (b) TiO_2_ NRs, and (c) PDIEH thin film coated on FTO and TiO_2_/PDIEH NHs. The Kubelka–Munk plots of (d) PDIEH in CHCl_3_, (e) TiO_2_ NRs, (f) PDIEH thin film coated on FTO, and (g) TiO_2_/PDIEH NHs.

The absorption spectrum of PDIEH recorded in chloroform showed multiple peaks located at 429 nm, 457 nm, 489 nm, and 525 nm (Fig. 2a) which match the reported spectrum.^42,43^ The absorption bands at 457, 489, and 525 nm are attributed to the 0–0, 0–1, and 0–2 electronic transitions, respectively.^42^ The spectral intensity of the 0–0 transition is smaller than that of the 0–1 transition. This might be due to the existence of a face-to-face-stacked dimer of PDI in the solvent.^42^ PDIEH showed maximum absorbance at 525 nm, which corresponds to the vibronic progression of the first S_0_–S_1_ transition, making PDIEH a good candidate as an organic semiconductor to be blended with TiO_2_ for PEC application.^44^ TiO_2_ barely absorbs any light in the visible spectrum, as shown in Fig. 2b. Fig. 2c displays the absorption spectra of PDIEH thin films on FTO (FTO/PDIEH) and TiO_2_/PDIEH NHs. TiO_2_/PDIEH NHs exhibited absorption in wide visible regions corresponding to the absorbance of PDIEH. FTO/PDIEH shows higher absorption intensity compared to TiO_2_/PDIEH NHs because of the higher concentration of PDIEH in FTO/PDIEH than in TiO_2_/PDIEH NHs. The absorption spectra of TiO_2_/PDIEH NHs were also recorded when changing the thickness of PDIEH on TiO_2_ (Fig. S6†). On increasing the thickness of PDIEH, no absorption is observed because it blocks the incident beam of the UV-vis spectrophotometer and no absorption is observed by the detector. The photoluminescence (PL) spectrum of PDIEH in CHCl_3_ exhibited emission peaks at 609 nm and 635 nm (Fig. S7a†). The PL spectrum for TiO_2_ NRs (Fig. S7b†) shows an emission peak at 413 nm arising from the radiative recombination of self-trapped excitons localized within the octahedral TiO_6_ matrix and oxygen vacancies (V_O_).^45^ The peak at about 441 nm is due to V_O_ with two trapped electrons, and the peaks at 469 nm and 483 nm are due to V_O_ with a single trapped electron center.^22,46^ The peak at 527 nm is ascribed to oxygen vacancy-related trap states.^22^ The PL spectra of PDIEH thin film on FTO (FTO/PDIEH) and TiO_2_/PDIEH NHs are shown in Fig. S7c.† TiO_2_/PDIEH NHs exhibit emission around 645 nm corresponding to the PDIEH as revealed from the emission spectrum of FTO/PDIEH. The Kubelka–Munk plots of PDIEH (Fig. 2d), TiO_2_ NRs (Fig. 2e), FTO/PDIEH (Fig. 2f), and TiO_2_/PDIEH NHs (Fig. 2g) were used to calculate their respective optical band gaps (*E*_g_). The *E*_g_ calculated for PDIEH, TiO_2_ NRs, FTO/PDIEH, and TiO_2_/PDIEH NHs were 2.28 eV, 3.2 eV, 2.05 eV, and 1.87 eV, respectively.

**Fig. 3:**
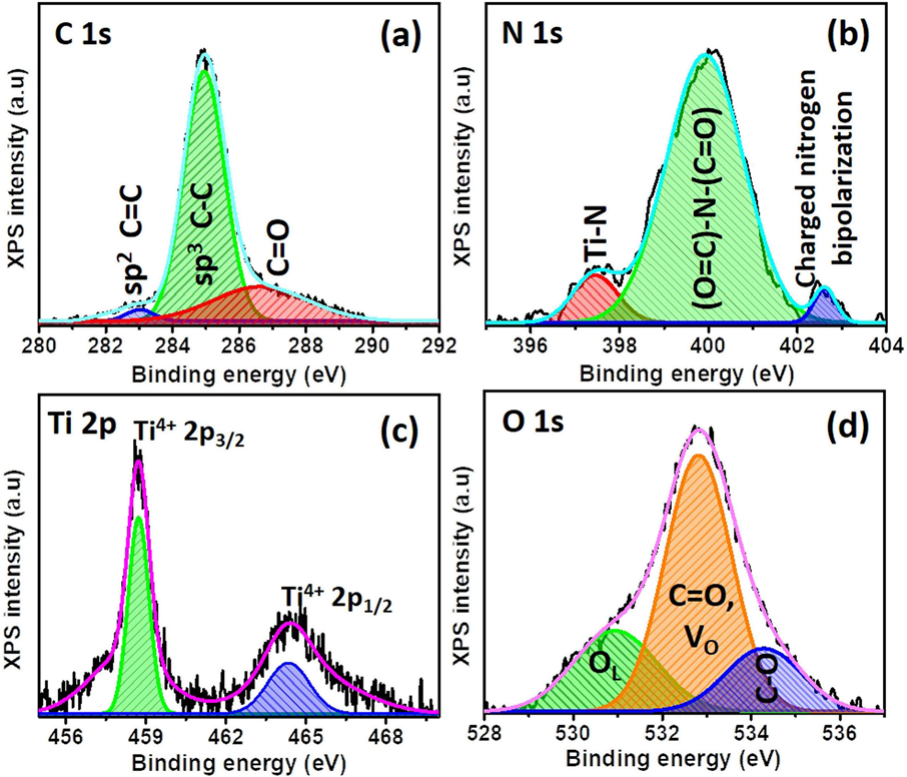
High-resolution peak-fitted XPS spectra of (a) C 1s, (b) N 1s, (c) Ti 2p, and (d) O 1s for TiO_2_/PDIEH NHs.

XPS was carried out to analyze the constituent elements of TiO_2_ (Fig. S8†) and TiO_2_/PDIEH NHs (Fig. 3). Fig. 3a shows that the XPS spectrum of C 1s for the NHs consists of three peaks at 283 eV, 284.9 eV, and 287.11 eV. The small peak at 283 eV is a signature of sp^2^ hybridized C=C carbon.^47,48^ The 284.9 eV peak corresponds to benzenic and adventitious carbon.^47^ The 288.8 eV peak corresponds to the carbon in the carbonyl group (C=O).^49^ The high-resolution XPS spectrum of N 1s (Fig. 3b) consists of three peaks centered at 402.96 eV, 400.51 eV, and 397.53 eV. The high binding energy 402.96 eV peak is attributed to the X-ray oxidized charged nitrogen bipolarity.^47^ The 400.51 eV peak is assigned to the imide ((O=C)–N–(C=O)) functional group.^50^ The 397.93 eV binding energy peak is related to the nitrogen involved in Ti–N bonds.^47^ The Ti 2p XPS spectrum (Fig. 3c and S8a†) consists of two doublet peaks centered at 459 eV and 464.73 eV representing the Ti 2p_1/2_ and Ti 2p_3/2_ states of Ti^4+^.^51^ The XPS of Ti 2p for both TiO_2_ and TiO_2_/PDIEH reveals the same type of Ti cluster in both materials. The O 1s spectrum (Fig. S8b†) of TiO_2_ depicts a 530.11 eV peak, corresponding to lattice oxygen (O_L_) in the TiO_2_ matrix. The secondary peak centered at 532.21 eV corresponds to oxygen vacancies (V_O_).^47,49^ The XPS of O 1s from TiO_2_/PDIEH NHs (Fig. 3d) shows small shifts in the O_L_ peak (530.91 eV) and V_O_ peak (532.81 eV) which might be due to the interaction of PDIEH with TiO_2_, as the peak centered at 532.81 eV is attributed to the carbonyl (C=O) group present in the organic compound. Moreover, the small peak at 534.32 eV corresponds to the C–O group occurring in PDI due to the resonance phenomenon in the imide bond.^49^ The above discussion reveals that there is covalent interaction between TiO_2_ NRs and PDIEH.

## 3. Photoelectrochemical (PEC) studies

**Fig. 4:**
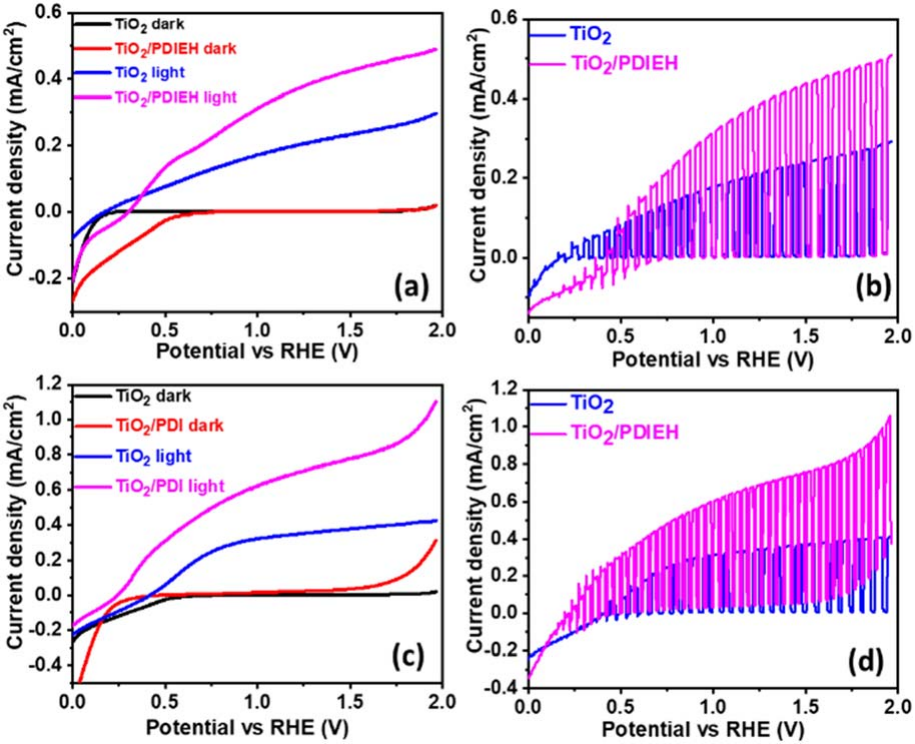
(a) The LSV plots in 0.5 M KOH solution. (b) The LSV plots under fast-chopped light in aq. 0.5 M KOH solution. (c) The LSV plots in aq. 0.5 M KOH + aq. 0.5 M urea solution. (d) The LSV plots under fast-chopped light in 0.5 M KOH + 0.5 M urea solution.

All PEC measurements of TiO_2_ NR and TiO_2_/PDIEH NH photoanodes were conducted in a three-electrode system on a CHI 660D electrochemical workstation (details are given in ESI†). Note that to convert the potential value (*V*_Ag/AgCl_) measured against the Ag/AgCl reference electrode into the reversible hydrogen electrode (RHE), eqn (S1)† was employed. Fig. 4a shows the linear sweep voltammetry (LSV) graphs in aq. 0.5 M KOH solution. The TiO_2_ NRs and TiO_2_/PDIEH NHs exhibit photocurrent densities (*J*_ph_) of 0.29 mA cm^−2^ and 0.48 mA cm^−2^ at 1.965 V_RHE_. This increment in *J*_ph_ after the formation of NHs is due to fast charge transmission at the electrode/electrolyte surface.^49^ A thin layer of PDIEH facilitates the absorbance of the photon within a wide-range of visible light that results in better charge separation. The PEC water oxidation onset potential is the potential in the LSV plot recorded in KOH solution at which the slope of *J*_ph_ meets the dark current.^51^ The water oxidation onset potentials shown by the TiO_2_ NR and TiO_2_/PDIEH NH photoanodes are 0.20 V_RHE_ and 0.30 V_RHE_, respectively (Fig. 4a) and exhibit anodic shifts of 0.1 V in the onset potential of the TiO_2_/PDIEH NH photoanode compared to the TiO_2_ NRs. It is worth mentioning here that, despite having an anodically shifted onset value compared to the TiO_2_ NR photoanode, the TiO_2_/PDIEH NH photoanode has a significantly higher *J*_ph_ than the TiO_2_ NR photoanode. The anodically shifted onset value for the TiO_2_/PDIEH NH photoanode suggests sluggish oxidation kinetics at a lower potential, which is probably attributable to the unfavorable surface properties toward PEC water oxidation arising from surface charge recombination at lower potential.^52^Fig. 4c shows the LSV graphs in KOH solution with urea. The TiO_2_ NRs and TiO_2_/PDIEH NHs exhibit *J*_ph_ of 0.42 mA cm^−2^ and 1.1 mA cm^−2^ at 1.965 V_RHE_. This increment in *J*_ph_ of both photoanodes in the presence of urea is due to UOR over the photoanodes.^53^ The fast-chopped light illumination LSV curve was recorded in KOH solution without (Fig. 4b) and with urea (Fig. 4d), confirming the fast and reproducible light sensitivity of the photoanodes. The PEC urea oxidation onset potential (*V*_op_) is the potential in the LSV plot at which the slope of *J*_ph_ meets the dark current.^54^ A PEC system with high *J*_ph_ and low *V*_op_ exhibits higher efficiency toward UOR.^55^ From the LSV graph of TiO_2_ NRs and TiO_2_/PDIEH in KOH solution with urea, the *V*_op_ for TiO_2_ NRs is found to be 0.40 V_RHE_, which is higher than the standard PEC urea oxidation *V*_op_ (0.37 V_RHE_).^56^ This higher *V*_op_ of TiO_2_ NRs towards PEC UOR reveals its lover activity towards PEC UOR. The *V*_op_ for TiO_2_/PDIEH NHs was found to be 0.24 V_RHE_, which is lower than the standard PEC urea oxidation *V*_op_ (0.37 V_RHE_).^56^ TiO_2_/PDIEH NHs exhibit a cathodic shift of 0.2 V compared to TiO_2_ NRs, indicating improved photocatalytic activity towards UOR after forming NHs. The improvement in the performance after forming NHs is due to the active surface, which allows fast charge transmission at the electrode/electrolyte surface.^49^ From the results, it can be estimated that TiO_2_/PDIEH NHs is not an appropriate catalyst for the water oxidation reaction, while TiO_2_ NRs is not an appropriate catalyst for UOR.^57^ The thickness of the semiconductor layer in type II NHs is of great importance to understanding the electron transfer, which is important for optimizing the efficiency of the PEC system. The effect of changes in coating thickness on the PEC response of NHs was studied. Fig. S9a† shows the LSV plots of TiO_2_/PDIEH NHs with variable thicknesses of the PDIEH layer which reveal that with increasing PDIEH thickness, the *J*_ph_ decrease. This decrease in *J*_ph_ is observed because with the increasing thickness, the exposure of the TiO_2_ to light decreases and thus the photogeneration of electron–hole pairs decreases. Additionally, this thick layer lacks the proper inter-junction contact between semiconductors which decreases the better charge transfer between NH layers.^58^

**Fig. 5:**
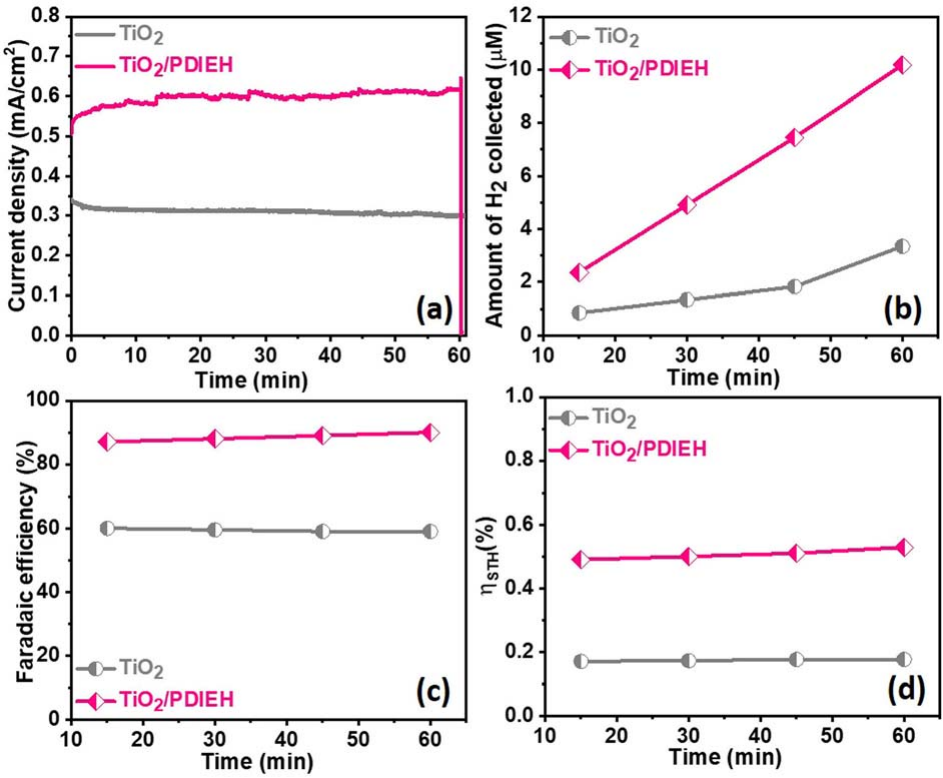
(a) *i*–*t* plots recorded at 0.96 V_RHE_ in 0.5 M KOH + 0.5 M urea solution. (b) The amounts of H_2_ gas collected at different time intervals. (c) Faradaic efficiency plots. (d) Solar-to-hydrogen (STH) conversion efficiencies.

The chronoamperometry (*i*–*t*) experiments of TiO_2_ NRs and TiO_2_/PDIEH NHs reveal that both photoanodes have good photostability (Fig. S9b†). In the *i*–*t* experiments of TiO_2_/PDIEH NHs, a gradual increase in the photocurrent density at the initial stage is observed which may be related to some surface phenomenon like rapid charge transfer at the electrode/electrolyte interface in the initial stages. FT-IR spectra of TiO_2_/PDIEH NHs (Fig. S10†) before usage and after 10 hours of the PEC stability test also confirm the stability of TiO_2_/PDIEH NHs. After the stability test, FT-IR spectra reveal that the structure of PDIEH has not changed. In PEC water oxidation, O_2_ gas is generated over the anode. This experiment was performed in 0.5 M KOH solution. Fig. S12a† shows the quantities of O_2_ collected using the TiO_2_ NR and TiO_2_/PDIEH NH photoelectrodes. The amounts of oxygen gas collected for the TiO_2_ NR and TiO_2_/PDIEH NH electrodes are 1.9 μmol cm^−2^ and 4.55 μmol cm^−2^, respectively. Both photoanodes exhibited a stable O_2_ generation ability. The faradaic efficiencies (FE) (details are given in ESI†) calculated for each photoelectrochemical OER are 55% and 81.3% for the TiO_2_ NR and TiO_2_/PDIEH NH electrodes, respectively (Fig. S12b†). The increment in the oxygen production in TiO_2_/PDIEH NH electrodes could be attributed to the nanoheterostructure formation. The H_2_ production capabilities of the photoanodes were tested by running an *i*–*t* experiment under constant light irradiation for 60 min (Fig. 5a). In PEC UOR, H_2_ was generated at the cathode. The amount of H_2_ generated was measured using an inverted-burette technique (Fig. S6a†). Fig. 5b shows the quantity of H_2_ collected after each 15 min at 0.96 V_RHE_. The quantities of H_2_ collected after 60 min for the TiO_2_ NR and TiO_2_/PDIEH NH electrodes are 3.32 μmol cm^−2^ and 10.2 μmol cm^−2^, respectively. The generation of H_2_ over the Pt wire electrode with the TiO_2_/PDIEH photoanode can be seen in Video S1.† The calculated FE for the TiO_2_ NR and TiO_2_/PDIEH NH electrodes are 58.85% and 90.21%, respectively (Fig. 5c). The FE of TiO_2_ is almost half that of TiO_2_/PDIEH NHs because the recombination rate of the photogenerated electrons and holes is high over TiO_2_,^25,59,60^ which reduces the urea oxidation tendency of bare TiO_2_ (LSV and *i*–*t* stability studies), resulting in a decrease in H_2_ production on the cathode. This results in a lowering of the faradaic efficiency in the case of bare TiO_2_. Hence, blending the semiconductor with TiO_2_ is required to suppress the charge recombination rate. TiO_2_/PDIEH NHs cannot acquire 100% FE because, during PEC UOR, N_2_ and CO_2_ are produced as a product of UOR and, at the same time, O_2_ generated from urea assists in water oxidation in urea solution.^61^ The urea assists water oxidation and consumes some fraction of the photogenerated holes to generate O_2_; this generated O_2_ reacts again with generated H^+^ to form water.^62^ This inhibits the FE from reaching 100%. The PEC catalytic activity of the TiO_2_ NR and TiO_2_/PDIEH NH electrodes towards PEC UOR was also computed from their solar-to-hydrogen (STH) conversion efficiencies (*η*_STH_). The *η*_STH_ is the chemical (H_2_) energy generated against the incident light, calculated using eqn (S2).†^63^ The *η*_STH_ calculated for the TiO_2_ NR and TiO_2_/PDIEH NH electrodes are found to be 0.17% and 0.52%, respectively (Fig. 5d). All the above results explain that TiO_2_/PDIEH NHs have better PEC catalytic activity toward UOR compared to TiO_2_ NRs.

**Fig. 6:**
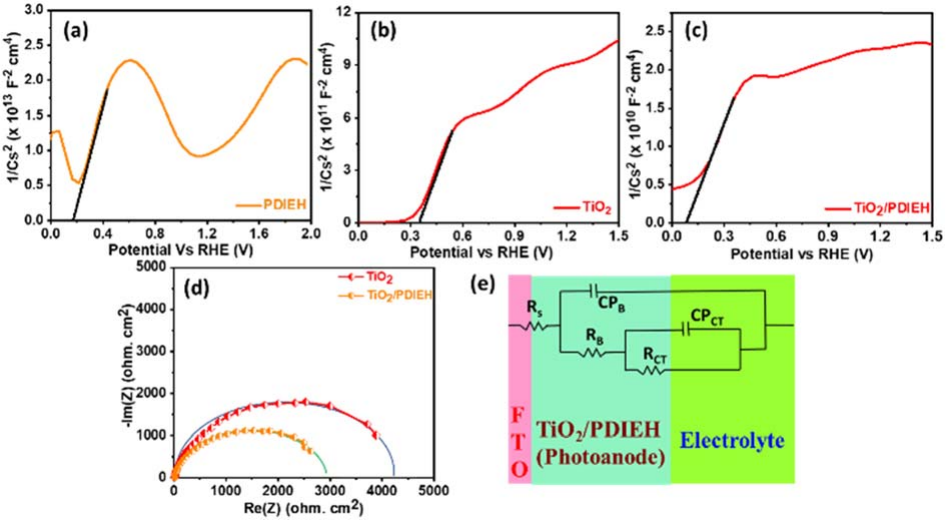
Mott–Schottky plots of (a) PDIEH, (b) TiO_2_ NRs, and (c) TiO_2_/PDIEH NHs. (d) Nyquist plots. (e) Equivalent circuit for TiO_2_ NRs and TiO_2_/PDIEH NHs. Here, all the experiments were recorded under visible light illuminated conditions in aq. 0.5 M KOH + aq. 0.5 M urea solution.

Mott–Schottky (M–S) plots for the PDIEH exhibit both negative and positive slopes *versus* the applied potential, revealing the simultaneous n-type and p-type nature of PDIEH (Fig. 6a).^64^ Considering the initial positive slope at a lower potential, n-type PDIEH shows a flat band potential (*V*_fb_) of 0.16 V_RHE_. The M–S plots of TiO_2_ NRs (Fig. 6b) and TiO_2_/PDIEH NHs (Fig. 6c) exhibit positive slopes, expressing their n-type nature.^49^ The decrease in the slope of TiO_2_/PDIEH compared to that of TiO_2_ is due to a significant increase in charge carrier density after forming NHs. The decrease in the M–S slope increases its charge donor density, resulting in enhancement of the electron charge concentration in the conduction band (CB).^47^ This enhancement of electrons in the CB moves the Fermi level towards the CB edge.^65^ Here, the *V*_fb_ for the TiO_2_ and TiO_2_/PDIEH electrodes are 0.35 V_RHE_ and 0.07 V_RHE_, respectively. The *V*_fb_ of an n-type semiconductor corresponds to the CB edge.^66^ The Nyquist plots of the photoanodes provide information about their charge transfer resistance (*R*_CT_) (Fig. 6d). *R*_CT_ is used to study the kinetics enhancement and separation efficiency of charge carriers of the electrodes.^53^ The equivalent circuit is used to explain the Nyquist plots (Fig. 6e). Since the charges generated in the photoanode undergo bulk recombination and surface recombination, an equivalent circuit with several resistors was used to distinguish them. *R*_s_ is the series resistance at the interface between FTO and the photoanodes, *R*_B_ is the bulk resistance in photoanodes, and CP_B_ is their capacity. Also, *R*_ct_ is the charge-transfer resistance at the interface between the photoanodes and electrolytes, and CP_ct_ is the constant phase element that represents the dielectrics of the electrical double layer at the electrode/electrolyte interfaces.^67^ The detailed values of resistance obtained from the fitted data of the equivalent circuit are given in Table S1.† The obtained resistance values reveal that, after the formation of the NHs, there is a decrease in the equivalent series resistance (*R*_S_), bulk resistance (*R*_B_), and *R*_CT_ values. The drop in *R*_B_ indicates a rise in the ion-conducting channels. The fall in *R*_CT_ indicates the fast and better charge transfer at an electrode–electrolyte interface in NHs.^68^

**Fig. 7:**
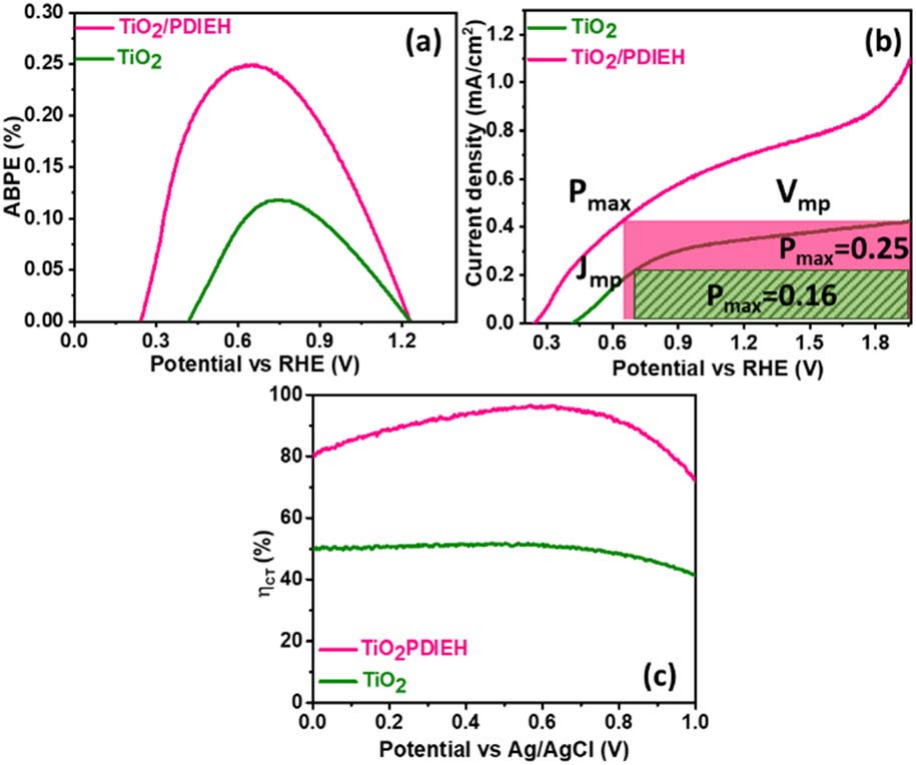
(a) The *η*% plots, (b) LSV *vs.* RHE plots, and (c) surface charge transfer efficiencies of TiO_2_ NRs and TiO_2_/PDIEH NHs.

For a quantitative analysis of the PEC UOR, the applied bias photon-to-current efficiency (ABPE) was calculated using eqn (S3).†^69^Fig. 7a exhibits the maximum ABPE (%) values of both photoanodes at different V_RHE_. TiO_2_ NRs exhibit 0.11% ABPE at 0.72 V_RHE_, while TiO_2_/PDIEH NHs show 0.25% ABPE at 0.63 V_RHE_. Compared to TiO_2_ NRs, the TiO_2_/PDIEH NHs exhibit higher ABPE due to the TiO_2_ NR being decorated with a visible-light active organic semiconductor which greatly improves the photoresponse under visible light. The ideal regenerative cell efficiency (*η*_IRC_) is calculated from the LSV plots in Fig. 7b (replotted from LSV plots in Fig. 3c) using eqn (S4).† The maximum power densities (*P*_max_) for TiO_2_ NRs and TiO_2_/PDIEH are indicated by the green and pink shaded areas, respectively. The *η*_IRC_% values calculated for TiO_2_ and TiO_2_/PDIEH are 0.16% and 0.25%, respectively. Interestingly, the obtained *η*_IRC_% and *η*% values of each photoanode are quite comparable. Table S2† compares the TiO_2_/PDIEH NHs' UOR performance to a few recently published UOR results. This comparison shows that TiO_2_/PDIEH NHs is a better photocatalyst than those reported in the past. LSV measurements for the photoelectrodes were also carried out in 0.5 M aq. Na_2_SO_3_ to understand the enhanced surface charge transfer efficiency (*η*_CT_). Na_2_SO_3_ acts as a hole scavenger and helps to investigate the *η*_CT_. Owing to the low activation energy and fast kinetics for the oxidation of SO_3_^2−^ species, the *η*_CT_ for SO_3_^2−^ oxidation can be presumed to be 100%.^70,71^*η*_trans_ is calculated using eqn (1),^71^ where 
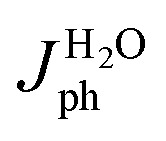
 and 
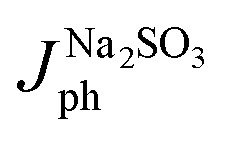
 are the photocurrent densities obtained in 0.5 M aq. KOH and 0.5 M aq. Na_2_SO_3_, respectively.1
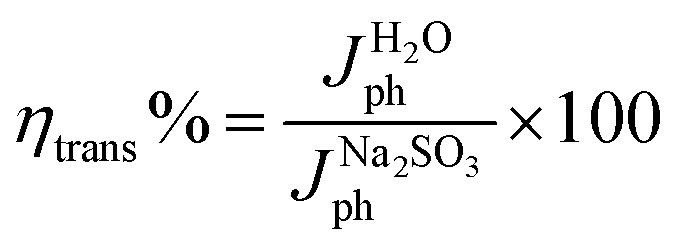


Fig. 7c displays that the TiO_2_ NR photoanode shows a maximum *η*_CT_ value of ∼51%, whereas the TiO_2_/PDIEH NH photoanode exhibits a higher maximum *η*_CT_ value of ∼96.99%.

**Fig. 8:**
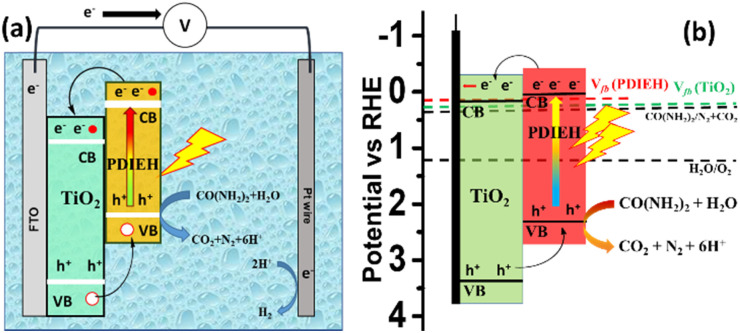
(a) PEC cell diagram with expected charge flow in the TiO_2_/PDIEH NH photoanode for UOR. (b) The energy diagram based on the *V*_fb_.

Fig. 8a shows the PEC cell diagram with expected charge flow in the TiO_2_/PDIEH NH photoanode for solar-driven UOR and H_2_ generation. The following equations describe the chemical reactions at the electrodes.^72,73^Anode: CO(NH_2_)_2_ + H_2_O → 6H+ + CO_2_ + N_2_, *E*_0_ = 0.37 V_RHE_Cathode: 6H^+^ + 6e^−^ → 3H_2_, *E*_0_ = 0 V_RHE_Total: CO(NH_2_)_2_ + H_2_O → CO_2_ + N_2_ + 3H_2_, *E*_0_ = 0.37 V_RHE_

In this PEC cell, TiO_2_/PDIEH forms a type-II heterojunction with a staggered gap. Upon light irradiation, TiO_2_/PDIEH is excited by gaining photons, resulting in the photogeneration of electron (e^−^) hole (h^+^) pairs in the CB and valence band (VB) of PDIEH, respectively. As the CB of PDIEH is located at a higher energy level than that of TiO_2_, the photogenerated e^−^ in the CB of PDIEH transfers to the CB of TiO_2_. At the same time, the h^+^ travels in the reverse direction, *i.e.*, from the VB of TiO_2_ to the VB of PDIEH. The photogenerated h^+^ in the VB of PDIEH is positive enough to carry out UOR to produce CO_2_, N_2_, and H^+^.^74,75^ The e^−^ in the CB of TiO_2_ moves rapidly at the Pt electrode and reduces H^+^ to produce H_2_. The working principle of the TiO_2_/PDIEH NHs is illustrated by the energy diagram (Fig. 8b) showing the e^−^ transfer from the CB of PDIEH to the CB of TiO_2_. The band diagram is constructed from the obtained *V*_fb_ and *E*_g_ values of PDIEH and TiO_2_. The conduction band potential (*V*_CB_) of TiO_2_ was measured as 0.25 V_RHE_ (assuming 0.1 negatives of its *V*_fb_ (0.35 V_RHE_))^76^ and the valence band potential (*V*_VB_) of TiO_2_ was obtained as 3.45 V_RHE_ from the *E*_g_ value (3.2 eV). Similarly, the *V*_CB_ of PDIEH is considered to be 0.06 V_RHE_ (assuming 0.1 negatives of its *V*_fb_ (0.16))^76^ and the *V*_VB_ of PDIEH was obtained as 2.34 V_RHE_, considering the *E*_g_ value (2.28 eV).

## 4. Conclusions

In summary, an inorganic/organic nano-heterostructure was designed and synthesized by coating the surface of TiO_2_ nanorods with a thin layer of PDIEH *via* the spin-coating technique. The spin-coating method established a PDIEH coating of uniform thickness. The resultant TiO_2_/PDIEH NHs as photoanodes were responsive to visible-light illumination and demonstrated an ultrahigh *J*_ph_ of 1.1 mA cm^−2^ at 1.96 V_RHE_ compared to that of TiO_2_ NRs derived from PEC urea oxidation. TiO_2_/PDIEH NHs exhibit a PEC urea oxidation onset potential of 0.24 V_RHE_, which is very low compared to that of the standard PEC *V*_op_ (0.37 V_RHE_). The combination of PDIEH and TiO_2_ NRs dramatically improved the PEC performance by enhancing light absorbance. The TiO_2_/PDIEH NHs also have high PEC stability under continuous light irradiation. Engineering these inorganic/organic NHs by taking advantage of the high electrical conductivity of the TiO_2_ NRs and the structural flexibility of the PDIEH gives rise to increased activities and is a promising strategy for the systematic design of the next generation of UOR photoelectrocatalysts.

## Author contributions

J. B. – conducted all experiments and writing, D. M. S. – data analysis, reviewed, and editing. A. V. M.-reviewed and edited, P. T. B. – contributed to some of the data analysis, H. S. K. – data analysis and editing, and SSZ – proposed and supervised the whole project, funding acquisition, and data analysis.

## Conflicts of interest

There are no conflicts to declare.

## Supplementary Material

NA-005-D3NA00294B-s001

NA-005-D3NA00294B-s002
