# Supplementary material for: A TiO2 nanorod and perylene diimide based inorganic/organic nanoheterostructure photoanode for photoelectrochemical urea oxidation
Source: Nanoscale Adv. 2023 Nov 9;5(23):6670–7. doi: 10.1039/d3na00294b (PMC10662079; doi:10.1039/d3na00294b)
Supplement: NA-005-D3NA00294B-s001 [file NA-005-D3NA00294B-s001.pdf]

Electronic Supplementary Material (ESI) for Nanoscale Advances.
This journal is © The Royal Society of Chemistry 2023

ARTICLE

TiO_2 nanorods and perylene diimide based inorganic/organic nanoheterostructure photoanode for photoelectrochemical Urea oxidation

Ms. Jasmine Bezboruah[#], Mr. Devendra Mayurdhwaj Sanke[#], Dr. Ajay Vinayakrao Munde, Ms. Palak Trilochand Bhattad, Dr. Himadri Shekhar Karmakar, and Sanjio S. Zade*

Department of Chemical Sciences and Centre for Advanced Functional Materials, Indian Institute of Science Education and Research (IISER) Kolkata, Mohanpur, Nadia 741246, West Bengal, India.

Corresponding Authors: S. S. Zade (sanjiozade@iiserkol.ac.in).

Supporting information list

1) Experimental section:

- **Materials and reagents for inorganic synthesis.**
- **Materials and reagents for organic synthesis.**
- **Synthesis of TiO_2 nanorods (TiO_2 NRs).**

2) Synthesis of Organic Semiconductor

3) Preparation of TiO_2/PDIEH

4) Material characterization.

- **Photoelectrochemical measurements.**

5) Supplementary results.

- **PL spectrum.**
- **FTIR**
- **Photoelectrochemical setup**

Comparison of the performances of the present electrodes with the other hybrid photoanodes

References.

Please do not adjust margins

1) Experimental section:

Materials and reagents for synthesis

Fluorine-doped tin oxide (FTO)-coated glass substrate (~7 Ohm/sq. Sigma-Aldrich), Titanium (IV) butoxide ($Ti(OBu)_4$, Sigma-Aldrich, 97%), Hydrochloric acid (HCl 37%, Merck India), de-ionized (DI) water (Merck Millipore). Perylene-3,4,9,10-tetracarboxylic dianhydride;(PDA) (97% assay); 2-ethylhexylamine (98%); Imidazole (99% assay); Zinc Chloride (98%) were purchased from Sigma Aldrich. Chloroform ($CHCl_3$) was bought from Merck India. All these chemicals were used without further purification.

Synthesis of TiO_2 NRs

At first, FTO substrates (1/2 cm^2) were washed with distilled water to remove dust particles and cleaned with acetone, ethanol, and isopropanol, respectively, in an ultrasonic bath for 30 min and then dried under an IR lamp. In a beaker, 8 ml ultrapure Milli-Q water was taken, and to it, 8 ml HCl was added, and the solution was stirred for 10 min. Then to it, 0.3 ml of $Ti(OBu)_4$ was added dropwise into the solution under the stirring condition, and the solution was kept stirring for another 20 min. The clean FTO substrates were placed tilted against the wall of a 25 ml Teflon-lined stainless steel autoclave with the conducting surface facing upward and filled with a suitable amount of the precursor solution. Finally, the sealed autoclave was inserted inside a hot air oven and heated at 150 °C for 4 h. After completion of time, allow it to cool to room temperature naturally; after the oven reaches room temperature through natural cooling, the FTO substrates were taken out of the autoclave and washed with plenty of DI water, ethanol, and acetone, respectively. The as-prepared TiO_2 NRs samples were later annealed at 400 °C for 2 h in the air inside a muffle furnace with a ramp rate of 2 °C/min.

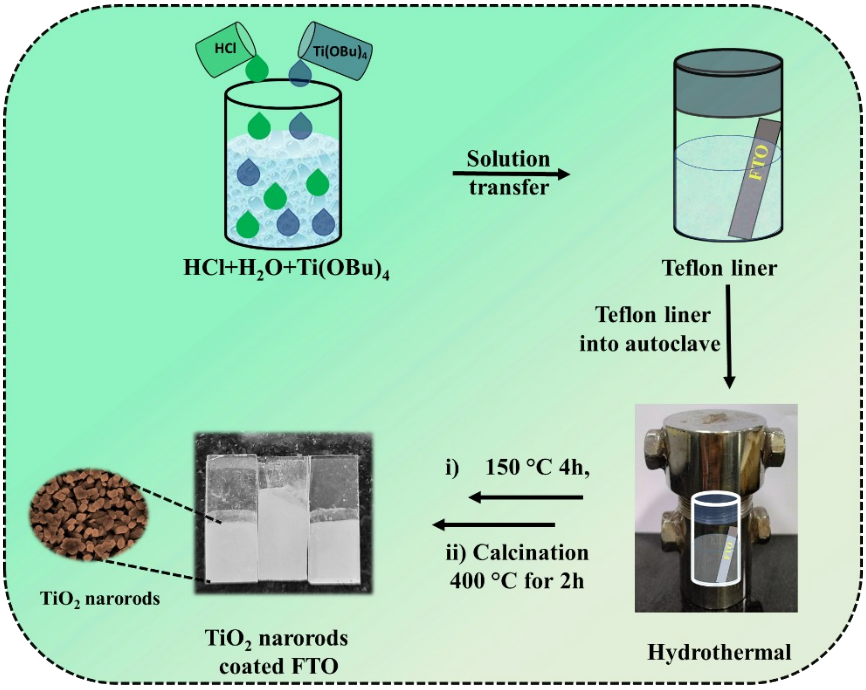

Figure S1. Schematic diagram of the synthesis of TiO_2 nanorods via hydrothermal process.

2) Preparation of Organic Semiconductor

Synthesis of N,N-bis(2-ethylhexyl) perylene-3,4,9,10-tetracarboxylic diimide (PDIEH)

PDIEH was synthesized according to a previously reported procedure from commercially available Perylene-3,4,9,10-tetracarboxydianhydride (PDA).[1] PDA (0.45 g, 1.14 mmol) was dissolved in 9.20 g of imidazole at 90 °C. Then, 0.44 mL (2.76 mmol) of 2-ethylhexylamine was added to the solution, and the mixture was warmed to 180 °C and stirred for 4 h. After cooling to room temperature, the solution was treated with 10 mL of water and then with 70 mL of 2 N HCl. The mixture was stirred for 12 h, and the resulting dark-red solid was filtered off and washed thoroughly with distilled water until the pH of the washings turned to neutral and dried. The crude product was purified by chromatography on silica gel using chloroform/ethylacetate (20:1) by volume as the eluent and a deep-red solid were recovered. ^1H NMR (500 MHz, Chloroform-d) δ 8.62 (s, 4H), 8.54 (s, 4H), 4.15 (s, 4H), 1.96 (s, 2H), 1.34 (s, 16H), 0.93 (s, 12H). IR(cm^{-1}): 3093 (νCH arom); 2922,2865 (νCH aliph); 1648,1597 (νCO imide); 1438,1349,1253,1170 (νCC ring).

This journal is © The Royal Society of Chemistry 20xx

Please do not adjust margins

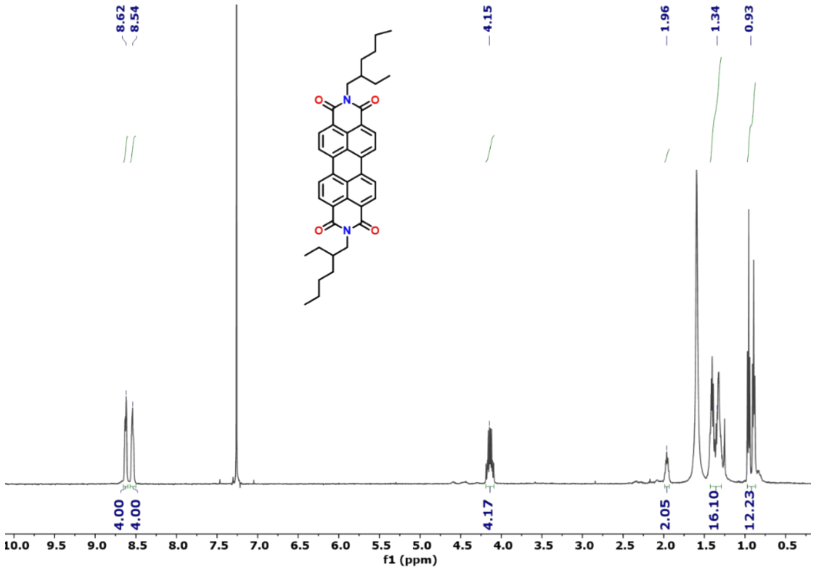

Figure S2: NMR Spectrum of PDIEH

3) Preparation of TiO_2/PDIEH NHs

For the preparation of TiO_2/PDIEH NHs, the spin-coating technique was used to fabricate a thin film of PDIEH over TiO_2. Firstly, the solution of compound PDIEH (10 mg), in $CHCl_3$ (1 ml) was prepared. The surface of the TiO_2 sample was drenched with PDIEH solution by drop-casting. Then the TiO_2 sample was spin at 3000 rpm for 15 s in a spin-coater. Finally, these samples were subjected to annealing for 1 h under nitrogen flow at 100 °C inside a conical flask with a standard joint neck, and placed inside a silicone oil bath.

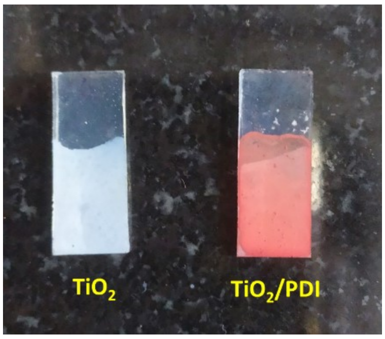

Figure S3: Image of FTO coated TiO_2 NRs (Left) and TiO_2/PDIEH NHs (Right).

4) Material characterization

A field emission electron microscope (FESEM, Zeiss SIGMA 300, and Zeiss SUPRA 55-VP) was used to study the morphology of the TiO_2 NRs and TiO_2/PDIEH NHs. UV-Vis-NIR absorption spectra of the materials were recorded at room temperature using an Agilent Cary 3500 spectrophotometer, whereas to record photoluminescence spectra of the materials at room temperature, a Horiba Fluoromax 4 spectrofluorometer (excitation wavelength 325 nm) was used. The Bruker spectrometer was used to record the nuclear magnetic resonance (NMR) spectrum of PDIEH with $CDCl_3$ as the solvent.

Photoelectrochemical measurements

Photoelectrochemical (PEC) measurements of TiO_2 NRs and TiO_2/PDIEH NHs photoanodes were conducted in a three-electrode system with a potentiostat (CH Instruments CHI 660D Electrochemical Workstation) under simulated AM 1.5G solar light irradiation, where a filtered Newport Xe lamp with a light intensity of 100 mW/cm^2 was used as the visible-light source. In a standard electrochemical cell (bought from CH Instruments), Ag/AgCl (saturated in 1 M aq. KCl) was used as the reference electrode, and the as-prepared electrodes were utilized as the working electrode, whereas a Pt wire was used as the counter electrode. The Nyquist plots of the two electrodes were obtained by performing the AC impedance measurement in the frequency range 1 MHz to 1 Hz at 0 $V_{Ag/AgCl}$ (with AC perturbation of 5 mV) under illuminated conditions. The linear sweep voltammetry (LSV) measurements were carried out at a 50 mV/s scan rate. An aqueous electrolyte containing 0.5 M KOH+0.5 M Urea was generally used. It is to be noted that to convert the potential value ($V_{Ag/AgCl}$) measured against the Ag/AgCl reference electrode into the reversible hydrogen electrode (RHE), the following Equation S1[2] is employed, where $V^0_{Ag/AgCl}$ = 0.1976 V at 298 K in saturated KCl.

Please do not adjust margins

$$V_{RHE} = V_{Ag/AgCl} + 0.0591 \times pH + V^{o}{}_{Ag/AgCl}$$ (Equation S1)

The quantity of H_2 collected and the photocurrent density observed during H_2 measurement are used to calculate the Faradaic efficiency (FE) The amount of hydrogen gas that evolved at the cathode during the overall urea oxidation reaction was estimated using the inverted-burette technique. The faradaic efficiencies for the HER were calculated using the following equation.

$$FE\% = \frac{experimentally\ obtained\ \mu mol\ of\ gas}{theoretically\ predicted\ \mu mol\ of\ gas} \times 100$$

Faraday's law is used to calculate the theoretical amount of gas, as given below.

$$n = \frac{I \times t}{z \times F}$$

Where *n* is the amount of gas (in mol), *I* is the current (in A), *t* is the time (in sec), *z* is the number of electrons involved (2 for H_2, 4 for O_2), and *F* is the Faraday constant (96485 $C.mol^{-1}$)

The performance of the catalyst towards PEC urea oxidation is also quantified by the solar to hydrogen conversion (STH) efficiency (η_{STH}), which is determined under standard solar irradiation generated with visible-light illumination (light intensity 100 mW/cm^2). The η_{STH} is defined as the amount of chemical (H_2) energy produced against the incident solar energy and can be determined using Equation S2,[3] where J_{ph} is photocurrent density, η_F is the Faradaic efficiency of H_2 production, and P_{in} is incident visible-light intensity (100 mW/cm^2).

$$\eta_{STH}(\%) = \left[\frac{J_{ph}(mA/cm^2) \times V_{RHE} \times \eta_F}{P_{in}(mW/cm^2)}\right]_{AM\ 1.5G}$$ (Equation S2)

The ABPE% is calculated using Equation S3,[4] where V_a is the applied potential recorded between the counter and the working electrode, P_o is the light intensity incident on the photoanode, and J_{ph} is the photocurrent density.

$$ABPE\% = \frac{J_{ph}(1.23 - V_a)}{P_o} \times 100$$ (Equation S3)

The η_{IRC} is calculated using Equation S4,[5] P_o represents the incident light intensity, V_{mp} and J_{mp} are both referenced to the applied potential (in RHE) and the photocurrent density, respectively, corresponding to the output power at the maximum power density (P_{max}). An IRC is analogous to solid-state PVCs, and therefore the same equation is used to express ideal regenerative efficiency as it is used to describe solid-state PVCs.

$$\eta_{IRC}\% = \frac{P_{max}}{P_o} \times 100 = \frac{(V_{mp} \times J_{mp}) \times 100}{P_o}$$ (Equation S4)

5) Supplementary results.

This journal is © The Royal Society of Chemistry 20xx

Please do not adjust margins

Please do not adjust margins

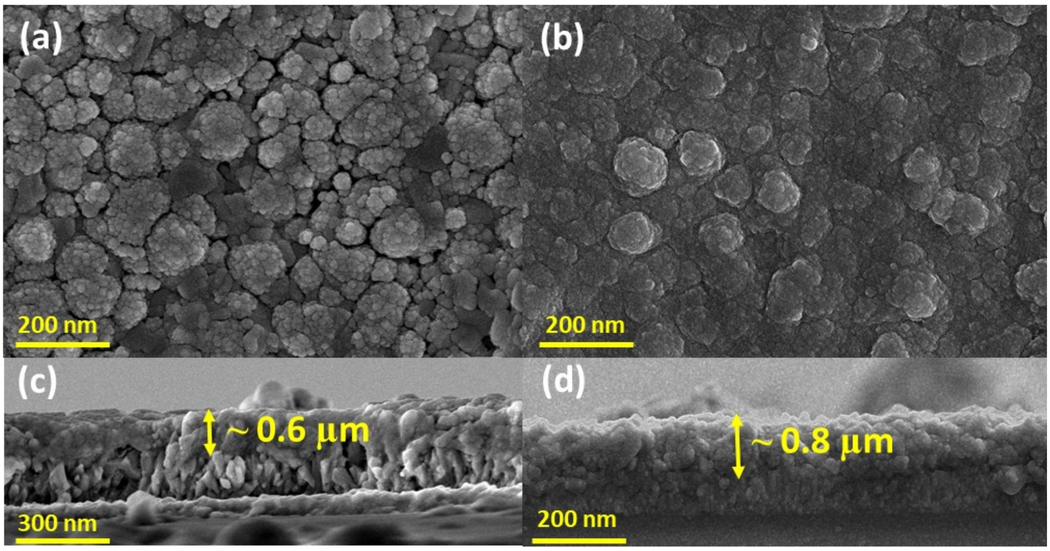

Figure S4: FESEM images of TiO_2/PDIEH NHs with (a) ~0.6µm and (b) ~0.8µm thickness of PDIEH. Cross-section FESEM image of TiO_2/PDIEH NHs with (a) ~0.6µm and (b) ~0.8µm thickness of PDIEH.

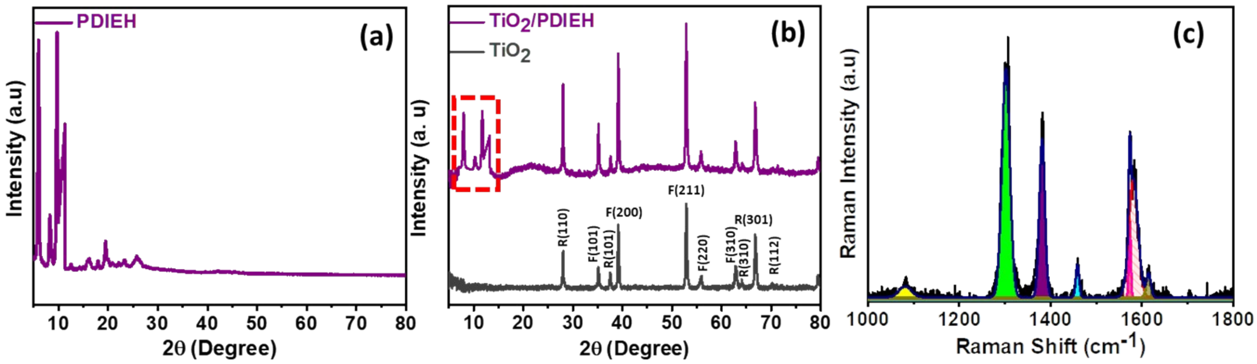

Figure S5: (a) XRD pattern of PDIEH powder and (b) XRD patterns of different materials. The (hkl) values with R and F represent the (hkl) values of rutile TiO_2. (c) Deconvolated Raman spectra of PDIEH.

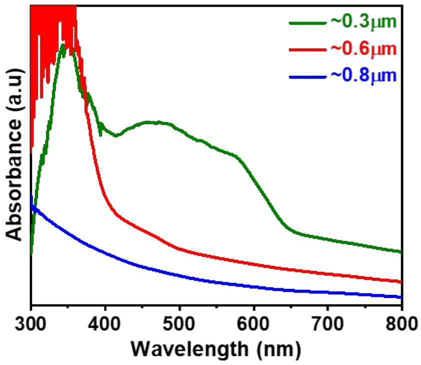

Figure S6: Absorption spectra of TiO_2/PDIEH NHs with ~0.3µm, ~0.6µm and ~0.8µm thickness of PDIEH

PL spectrum

Please do not adjust margins

Please do not adjust margins

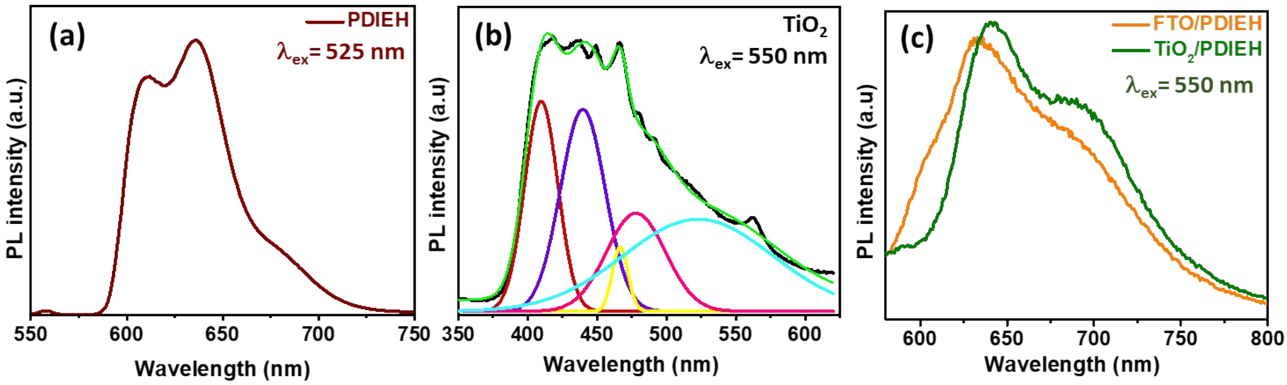

Figure S7: PL spectra of (a) PDIEH in $CHCl_3$, (b) TiO_2 NRs, and (c) PDIEH thin film coated on FTO and TiO_2/PDIEH NHs.

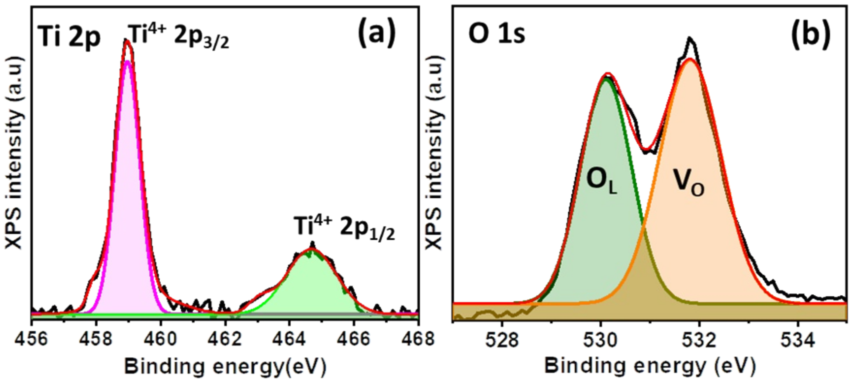

Figure S8: XPS of (a) Ti 2p and (b) O 1s of TiO_2 NRs

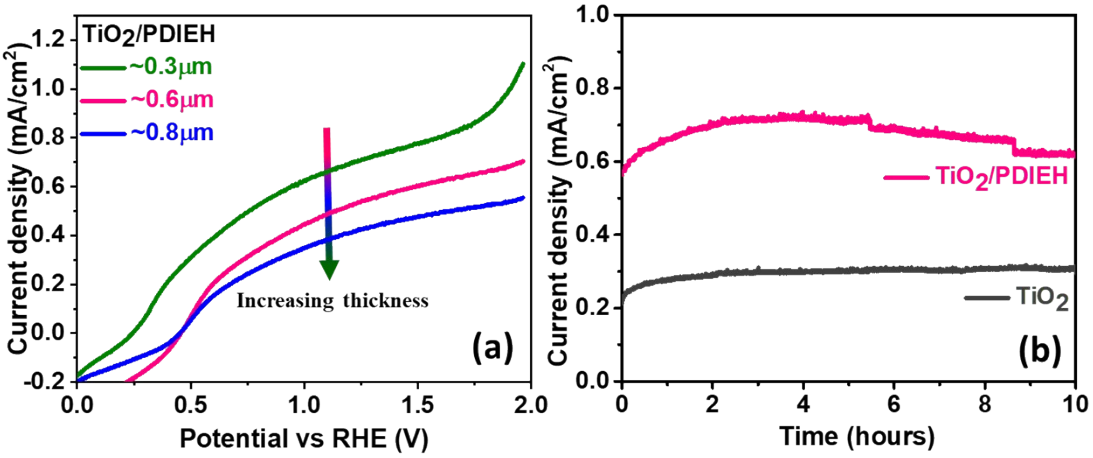

Figure S9: (a)) The LSV plots in aq. 0.5M KOH + aq. 0.5M Urea solution of TiO_2/PDIEH NHs with variable thickness of PDIEH. (b) i-t stability test for urea oxidation on TiO_2 NRs and TiO_2/PDIEH at an applied potential of 0.96 V_{RHE} in 0.5M KOH + 0.5M urea solution for 10 hours.

FTIR

This journal is © The Royal Society of Chemistry 20xx

Please do not adjust margins

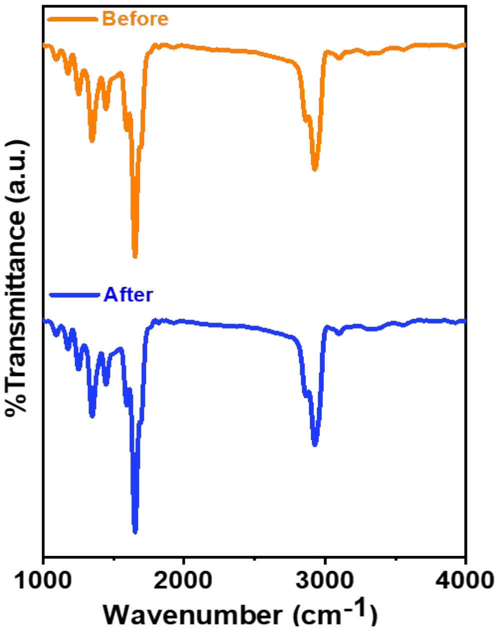

Figure S10. Fourier-transform infrared (FTIR) spectra of TiO_2/PDIEH NHs before use and after 1 h of photoelectrochemical stability test.

Photoelectrochemical setup

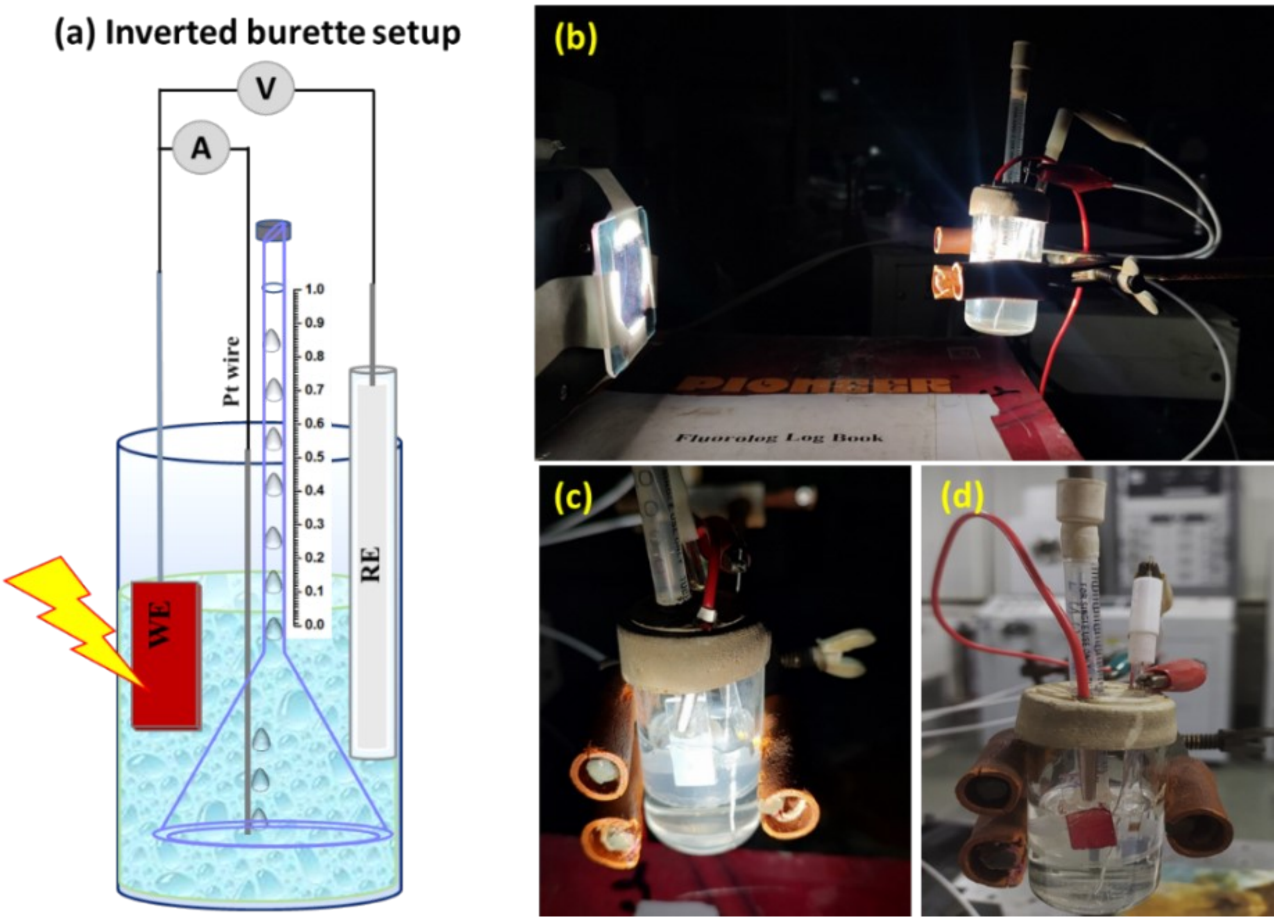

Figure S11: (a) Diagram of Inverted Burette Setup for H_2 Measurement (b) Picture of light illumination on Inverted Burette Setup. Picture of Inverted Burette Setup for H_2 measurement with (c) TiO_2 photoanode (d) TiO_2/PDIEH photoanode.

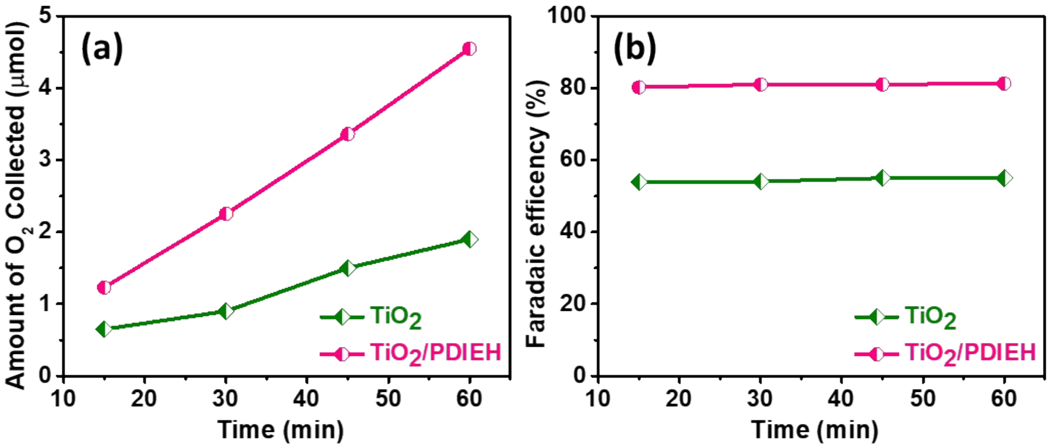

Figure S12: (a) Oxygen gas collected at different time intervals and (b) Photoelectrochemical OER Faradaic efficiency plot for the TiO_2 and TiO_2/PDIEH NHs photoanodes. Here experiments are carried out in 0.5M KOH solution

Nyquist plot fitted parameters from equivalent circuits.

Table S1. Nyquist plot fitted parameters from equivalent circuits.

 This journal is © The Royal Society of Chemistry 20xx

Resistance	TiO_2	TiO_2/PDIEH
Series resistance (R_s)	10.07Ω	8.3Ω
Bulk resistance (R_b)	20.16Ω	12.18Ω
Charge transfer resistance (R_{CT})	3867Ω	2792Ω

Comparison of the performances of the present electrodes with the other hybrid photoanodes

Table S2. PEC performances of some reported inorganic/organic NHs photoanodes.

Photoanode	Electrolyte	E_{onset} (V_{ref})	Stability Test Time	Light source and light intensity (mW/cm^2)	Ref.
Ni-TiO_2	0.1 M KOH<br>0.05 M urea Human urine	0.92 $V_{Ag/AgCl}$	50 hour	150 W xenon lamp AM 1.5 global filter. (100 mW cm^{-2})	[6]
Ni-TiO_2/p-NDINBT	0.5 M KOH<br>0.5 M urea	0.36 V_{RHE}	1 hour	Newport xenon lamp AM 1.5 global filter. (100 mW cm^{-2})	[7]
TiO_2–CdS–Ni	1 M NaOH<br>0.33 M urea	0.45 $V_{Ag/AgCl}$	NA	150 W xenon lamp AM 1.5 global filter. (100 mW cm^{-2})	[8]
Ni catalyst	1.0 M KOH<br>0.1 M urea	0.35 $V_{Ag/AgCl}$	10 min	Not mentioned	[9]
$La_4Ni_3O_{10}$	0.1 M KOH<br>0.5 M urea	1.54 V_{RHE}	10 hour	300 W xenon lamp AM 1.5 global filter. (100 mW cm^{-2})	[10]
TiO_2-NT/Fe_2O_3	0.1 M Na2SO4 buffered<br>0.05 M borax (pH = 9.2)<br>5 g dm^{-3} urea	0.9 V_{SCE}	-	300 W Osram Ultra-<br>Vitalux lamp. (80 mW cm^{-2})	[11]
FTO/Ti-Fe_2O_3/Ni	1M NaOH (pH = 13.6)<br>0.33 M urea	0.7 V_{RHE}		Newport LSH7320 solar simulator AM1.5G light<br>(1000 Wm^{-2})	[12]
La_2NiO_4	0.1 M KOH<br>0.5 M urea	1.35 V_{RHE}	8 hours	NIR light irradiation<br>(100 Wm^{-2})	[13]
$Ni(OH)_2$/Ti-Fe_2O_3	1 M NaOH<br>0.1 M urea	0.50 V_{RHE}	-	500 W xenon lamp<br>(100 Wm^{-2})	[14]
Fe_2O_3/$Ni(OH)_2$	1 M KOH<br>0.33 M urea	0.8 V_{RHE}	200 sec	500 W xenon lamp<br>(100 Wm^{-2})	[4]
Ni–Fe_2O_3/Ni	1.0 M KOH<br>0.1 M urea	1.43 V_{RHE}	-	500W/m^2 OSRAM spot lamp	[15]
Si/SiO_x/Ni/NMO	1 M KOH<br>0.33 M urea	0.9 V_{RHE}	5 hours	Solar Simulator (LS0106), AM 1.5 global filter. (100 mW cm^{-2})	[16]
**TiO_2/PDIEH**	**0.5 M KOH<br>0.5 M urea**	**0.24 V_{RHE}**	**10 hour**	**Newport xenon lamp AM 1.5 global filter<br>(100 mW cm^{-2})**	**This Work**

References

1 F. Donati, A. Pucci, C. Cappelli, B. Mennucci and G. Ruggeri, *J. Phys. Chem. B*, , DOI:10.1021/jp711193u.

Please do not adjust margins

2 D. M. Sanke, A. V. Munde, J. Bezboruah, P. T. Bhattad, B. R. Sathe and S. S. Zade, *Energy & Fuels*, , DOI:10.1021/acs.energyfuels.2c04377.

3 J. S. L. Jin Hyun Kim, Dharmesh Hansora, Pankaj Sharma, Ji-Wook Jang, *Chem. Soc. Rev.*, 2019, **48**, 1908–1971.

4 A. Karrab, L. Lecarme, J. C. Lepretre, A. Nourdine, J. Deseure and S. Ammar, *Appl. Phys. A*, 2022, **128**, 474.

5 H. S. Karmakar, A. Sarkar, N. G. Ghosh, D. M. Sanke, C. Kumar, S. Das and S. S. Zade, *Chemosphere*, 2022, **301**, 134696.

6 G. Wang, Y. Ling, X. Lu, H. Wang, F. Qian, Y. Tong and Y. Li, *Energy Environ. Sci.*, 2012, **5**, 8215.

7 J. Bezboruah, D. M. Sanke, A. V. Munde, S. Das, H. S. Karmakar and S. S. Zade, *Int. J. Hydrogen Energy*, 2023, **48**, 7361–7373.

8 R. Zhao, G. Schumacher, S. Leahy and E. J. Radich, *J. Phys. Chem. C*, 2018, **122**, 13995–14003.

9 F. Guo, K. Ye, M. Du, X. Huang, K. Cheng, G. Wang and D. Cao, *Electrochim. Acta*, 2016, **210**, 474–482.

10 Y. Tao, L. Chen, Z. Ma, C. Zhang, Y. Zhang, D. Zhang, D. Pan, J. Wu and G. Li, *Chem. Eng. J.*, 2022, **446**, 137240.

11 W. M. Omymen, J. R. Rogan, B. Z. Jugović, M. M. Gvozdenović and B. N. Grgur, *J. Saudi Chem. Soc.*, 2017, **21**, 990–997.

12 L. Rebiai, D. Muller-Bouvet, R. Benyahia, E. Torralba, M. L. Viveros, V. Rocher, S. Azimi, C. Cachet-Vivier and S. Bastide, *Electrochim. Acta*, 2023, **438**, 141516.

13 M. Xiong, Y. Tao, L. Fu, D. Pan, Y. Shi, T. Hu, J. Ma, X. Chen and G. Li, *Catalysts*, 2022, **13**, 53.

14 D. Xu, Z. Fu, D. Wang, Y. Lin, Y. Sun, D. Meng and T. feng Xie, *Phys. Chem. Chem. Phys.*, 2015, **17**, 23924–23930.

15 A. Karrab, R. Bensimon, D. Muller-Bouvet, S. Bastide, C. Cachet-Vivier and S. Ammar, *Appl. Phys. A*, 2022, **128**, 1067.

16 J. Dabboussi, R. Abdallah, L. Santinacci, S. Zanna, A. Vacher, V. Dorcet, S. Fryars, D. Floner and G. Loget, *J. Mater. Chem. A*, 2022, **10**, 19769–19776.

This journal is © The Royal Society of Chemistry 20xx

Please do not adjust margins
